# Minnelide combined with anti-ANGPTL3-FLD monoclonal antibody completely protects mice with adriamycin nephropathy by promoting autophagy and inhibiting apoptosis

**DOI:** 10.1038/s41419-023-06124-0

**Published:** 2023-09-09

**Authors:** Baowei Ji, Junchao Liu, Ye Yin, Hong Xu, Qian Shen, Jian Yu

**Affiliations:** 1 Department of Nephrology, Children’s Hospital of Fudan University, Shanghai, China; 2 Department of Traditional Chinese Medicine, Children’s Hospital of Fudan University, Shanghai, China

**Keywords:** Paediatric kidney disease, Preclinical research

## Abstract

Minimal change disease (MCD) is the common type of nephrotic syndrome (NS) in children. Currently, there is an urgent need to explore new treatments because of the significant side effects of long-term use of glucocorticoids and immunosuppressive drugs and the failure to reduce proteinuria in some patients. Angiopoietin-like protein 3 (Angptl3) is an essential target of NS, and anti-ANGPTL3-FLD monoclonal antibody (mAb) significantly reduces proteinuria in mice with adriamycin nephropathy (AN). However, some proteinuria is persistent. Minnelide, a water-soluble prodrug of triptolide, has been used for the treatment of glomerular disease. Therefore, the present study aimed to investigate whether minnelide combined with mAb could further protect mice with AN and the underlying mechanisms. 8-week-old C57BL/6 female mice were injected with 25 mg/kg of Adriamycin (ADR) by tail vein to establish the AN model. A dose of 200 μg/kg of minnelide or 20 mg/kg of mAb was administered intraperitoneally for the treatment. In vitro, the podocytes were treated with 0.4 μg/mL of ADR for 24 h to induce podocyte injury, and pretreatment with 10 ng/mL of triptolide for 30 min or 100 ng/mL of mAb for 1 h before ADR exposure was used to treat. The results showed that minnelide combined with mAb almost completely ameliorates proteinuria and restores the ultrastructure of the podocytes in mice with AN. In addition, minnelide combined with mAb restores the distribution of Nephrin, Podocin, and CD2AP and reduces the level of inflammatory factors in mice with AN. Mechanistically, minnelide combined with mAb could further alleviate apoptosis and promote autophagy in mice with AN by inhibiting the mTOR signaling pathway. In vitro, triptolide combined with mAb increases the expression of Nephrin, Podocin, and CD2AP, alleviates apoptosis, and promotes autophagy. Overall, minnelide combined with mAb completely protects the mice with AN by promoting autophagy and inhibiting apoptosis.

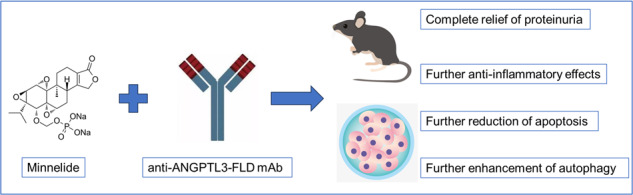

## Highlights

- Minnelide combined with mAb could completely relieve proteinuria in mice with AN.
- Minnelide combined with mAb could further alleviate apoptosis in mice with AN.
- Minnelide combined with mAb could further promote autophagy in mice with AN.
- Triptolide combined with mAb could further alleviate ADR-induced podocyte apoptosis.
- Triptolide combined with mAb could further promote autography in ADR-induced podocytes.

## Introduction

Minimal change disease (MCD) is the common type of nephrotic syndrome (NS) that accounts for up to 70–90% of NS in children >1 year of age [1]. The primary clinical manifestations are massive proteinuria, hypoalbuminemia, edema, and hyperlipidemia, with an incidence of about 2–7/100,000 [2, 3]. The etiology of MCD remains unclear, but immune dysregulation and podocyte modification play a critical role in disease development, with no alterations observed under light microscopy; the effacement of foot processes on electron microscopy was the major pathological feature [1, 2, 4].

The current first-line therapy for NS is oral glucocorticoids, achieving remission of proteinuria in 80–90% of children with NS; however, up to 80% of children experience relapses, and most will have to repeat glucocorticoids or receive additional immunosuppressive drugs to achieve remission of relapses [5–7]. Long-term glucocorticoid therapy can lead to growth impairment, obesity, cataracts, and Osteoporosis [8–10]. In addition, psychiatric and behavioral abnormalities such as anxiety, depression, and aggression are also very common [11]. Also, if proteinuria persists, kidney function will continue to decline, eventually leading to chronic kidney disease or end-stage renal disease [12, 13]. Therefore, exploring novel methods to treat NS while avoiding the side effects of drugs is an urgent requirement.

Angptl3 is a secreted glycoprotein with a molecular weight of 70 Kda [14]. Previous studies have shown that it is mainly involved in lipoprotein metabolism and angiogenesis [15–18]. However, its role in the kidney remains unclear. In a previous study, we found that Angptl3 is highly expressed in the kidney tissues of children with NS and puromycin or Adriamycin (ADR)-induced podocyte injury [19–21], and knockout of Angptl3 could largely alleviate proteinuria in mice with AN and protect against puromycin or adriamycin-induced podocyte damage [20, 22]. Mechanistically, ANGPTL3 activates the downstream FAK/PI3K/Rac1 signaling pathway by binding to podocyte-expressed integrin αVβ3 and mediating increased podocyte skeleton rearrangement and migration [22, 23]. Hence, we synthesized anti-ANGPTL3-FLD monoclonal antibody (mAb) by immunizing the mice with recombinant human ANGPTL3 protein and found that it significantly reduces proteinuria in mice with AN and puromycin-induced podocyte injury [24]. Conversely, the mAb alleviates proteinuria only to some extent in mice with AN, while substantial is still present. Therefore, the present study aimed to combine some drugs to seek a complete remission of nephropathy.

Triptolide, a major active ingredient extracted from the Chinese herb Tripterygium wilfordii Hook F (TWHF) [25], possesses anti-inflammatory, immunosuppressive, and anti-tumor properties [26–28]. However, the poor water solubility and bioavailability, as well as the reproductive toxicity from long-term use, severely limit the clinical application of the herb [26, 28]. Some studies have processed triptolide to obtain minnelide, a water-soluble prodrug of triptolide, which can also be metabolized into triptolide by the action of alkaline phosphatase in the body [26, 29]. Moreover, the reproductive toxicity of triptolide is time- and dose-dependent [27, 28, 30], rendering it safe to use minnelide in small doses for a short period.

Podocytes are terminally differentiated epithelial cells and major components of the glomerular filtration barrier [31]. Damage to the podocytes gives rise to proteinuria, a marker of most glomerular diseases [32, 33]. Autophagy plays a critical role in maintaining podocyte homeostasis and function [34, 35]. Autophagy dysfunction contributes to podocyte injury and promoting autophagy contributes to podocyte protection [36, 37]. The mTOR signaling pathway is closely related to podocyte autophagy, and activation of the pathway inhibits autophagy, leading to podocyte injury [38–40].

In the present study, we intraperitoneally injected both minnelide and mAb into mice with AN to investigate whether minnelide combined with mAb could achieve better protection compared to treatment with minnelide or mAb alone and its underlying protective mechanism. In vitro, triptolide combined with mAb was used to treat ADR-induced podocyte injury.

## Materials and methods

### Mice experiments

All mice experiments were approved by the Ethics Committee of Fudan University (Approval number: IDM2021055). A total of 25 mice were equally divided into five groups: Control, ADR, ADR+Minnelide, ADR+mAb, and ADR+Minnelide+mAb. The AN model was induced in 8-week-old C57BL/6 female mice by tail vein injection of 25 mg/kg of ADR (Sigma, D1515), while Control group was injected with an equivalent volume of saline through the tail vein. About 200 μg/kg.d of minnelide (Shanghai SCR-Biotech Co., Ltd) or once every 3 days of 20 mg/kg of anti-ANGPTL3-FLD mAb was injected intraperitoneally for the treatment of AN mice, while the non-treated group was injected with the same volume of saline intraperitoneally. At 1 week, mice were executed, and blood, urine, and kidney tissue were collected for subsequent experiments.

For the toxicity experiments of minnelide, 20 C57BL/6 female and male mice were divided into four groups separately, with five mice in each group, respectively: Control, 100 μg/kg.d, 200 μg/kg.d, and 400 μg/kg.d. An equivalent volume of saline was injected intraperitoneally for 1 week in the Control group, and 100, 200, and 400 μg/kg.d of minnelide was injected intraperitoneally for 1 week in the 100, 200, and 400 μg/kg.d groups, respectively. At 1 week, mice were executed to remove the liver, ovaries, and testes for hematoxylin-eosin (HE) staining.

### The production of anti-ANGPTL3-FLD monoclonal antibody

The method for the preparation of mAb is described in Ref [24]. In brief, the full-length ANGPTL3 protein and the ANGPTL3-FLD protein fragment were first successfully expressed using an Insect-Baculovirus expression system, and then a mouse anti-human monoclonal antibody targeting ANGPTL3-FLD was prepared using the classical hybridoma-monoclonal antibody technique.

### Blood and urine tests

The supernatants of blood and urine were obtained by centrifugation at 12,000 rpm for 15 min. Urine microalbumin and creatinine were measured using urine microalbumin and creatinine test kits, respectively. Serum albumin, triglycerides, and cholesterol were measured by the corresponding assay kits. The above kits were purchased from Nanjing Jiancheng Biotechnology Co., Ltd and operated according to the manufacturer’s instructions.

### Transmission electron microscopy (TEM)

Fresh kidney tissue of 1 mm^3^ size was removed and sequentially post-fixed, dehydrated at room temperature, osmotically embedded, polymerized, ultra-thinly sectioned, stained, and photographed under TEM (Hitachi, HT7700).

For the observation of the autophagosomes in the cytoplasm, the cells were collected by trypsinization and centrifugation at 1000 g for 5 min. The supernatant was discarded, followed by TEM examination as described above.

### Immunofluorescence staining

TUNEL kits (Servicebio, G1501) were used to detect apoptotic cells in paraformaldehyde-fixed kidney tissues, and the appropriate experimental protocols were applied. For immunofluorescence in tissues, paraffin sections were blocked with 10% donkey serum and incubated overnight at 4 °C with primary antibodies nephrin (1:100, Invitrogen, PA5-106921), podocin(1:100, Proteintech, 20384-1-AP), cd2ap(1:100, Invitrogen, PA5-51894), TNF-α(1:500, Servicebio, GB11188), IL-6(1:500, Servicebio, GB11117), IL-1β(1:300, Servicebio, GB11113), and LC3(1:500, Proteintech, 14600-1-AP), followed by secondary antibodies. Finally, the nuclei were restained with DAPI and observed under a confocal microscope (Olympus, FV3000).

For immunofluorescence in cells, each group of cells were given the corresponding treatment and sequentially subjected to cell rupture, serum closure, incubated with the corresponding primary antibodies nephrin (1:100, Invitrogen, PA5-106921), podocin (1:100, Proteintech, 20384-1-AP), cd2ap (1:100, Invitrogen, PA5-51894), and LC3(1:500, Proteintech, 14600-1-AP) overnight at 4 °C and secondary antibodies. Finally, after DAPI restaining nuclei, the cells were observed under confocal microscopy.

### Western blot

Kidney tissues and podocytes were first lysed with RIPA, followed by electrophoresis, membrane transfer, blocking, probing with primary antibodies nephrin (1:500, Invitrogen, PA5-106921), podocin (1:500, Proteintech, 20384-1-AP), cd2ap (1:500, Invitrogen, PA5-51894), Bax (1:500, Abcam, ab216494), Bcl-2 (1:500, Abcam, ab196495), Cleaved-caspase3 (1:500, Servicebio, GB11532), p-mTOR (1:1000,CST,5536 T), mTOR (1:1000,CST,2983 T), Beclin1(1:500, Servicebio, GB112053), p62(1:500, Servicebio, GB11239-1), and LC3(1:1000, Proteintech, 14600-1-AP) overnight at 4 °C, and incubation with secondary antibodies for 1 h at room temperature. Finally, the immunoreactive bands were developed by chemiluminescence and analyzed using Image J software.

### Enzyme-linked immunosorbent assay (ELISA) for the detection of inflammatory factors

TNF-α, IL-6, and IL-1β were detected in the serum of each group of mice using the corresponding ELISA kits, according to the manufacturer’s instructions.

### Cell culture and treatment

Mouse podocyte clone-5 (MPC-5) cells were cultured in RPMI-1640 medium containing 10% fetal bovine serum, 100 U/mL penicillin, 100 μg/mL streptomycin, and 10 U/mL murine interferon-gamma (IFN-γ) at 33 °C and transformed into the differentiated state by removing 10 U/mL murine (IFN-γ) in the culture at 37 °C for 10–14 days [41]. In addition, podocyte injury was induced by exposure to 0.4 μg/mL of ADR (Sigma, D1515) for 24 h after pretreatment with 10 ng/mL of triptolide (TCI, T2899) for 30 min or 100 ng/mL of mAb for 1 h. To investigate the protective effect of triptolide combined with mAb on ADR-induced podocyte injury, mouse podocytes were divided into five groups: Control, ADR, ADR+Triptolide, ADR+mAb, and ADR+Triptolide+mAb.

### Cell apoptosis detection

After treatment, mouse podocytes were trypsin digested, resuspended in phosphate-buffered saline (PBS), stained using the Annexin V-FITC Apoptosis Detection Kit (Beyotime, C1062L), and finally assayed using flow cytometry (BD, Biosciences).

### stubRFP-sensGFP-LC3 adenovirus transfection

MPC-5 cells were incubated on six-well plates and infected with the stubRFP-sensGFP-LC3 adenovirus (Shanghai Genechem Co., Ltd). Cells were infected for 72 h and used for subsequent experiments. The groups of treated cells were photographed under a confocal microscope (Olympus, FV3000) to monitor autophagy flux, and the number of red and yellow dots was counted.

### Statistical analysis

All experimental data from three independent replicates were analyzed by GraphPad Prism 9.0.0 (GraphPad Software, USA). Data were presented as mean ± SD, and differences between groups were compared using one-way ANOVA, with *p* < 0.05 considered statistically significant.

## Results

### Renal protective effect of minnelide combined with anti-ANGPTL3-FLD monoclonal antibody in mice with AN

**Fig. 1:**
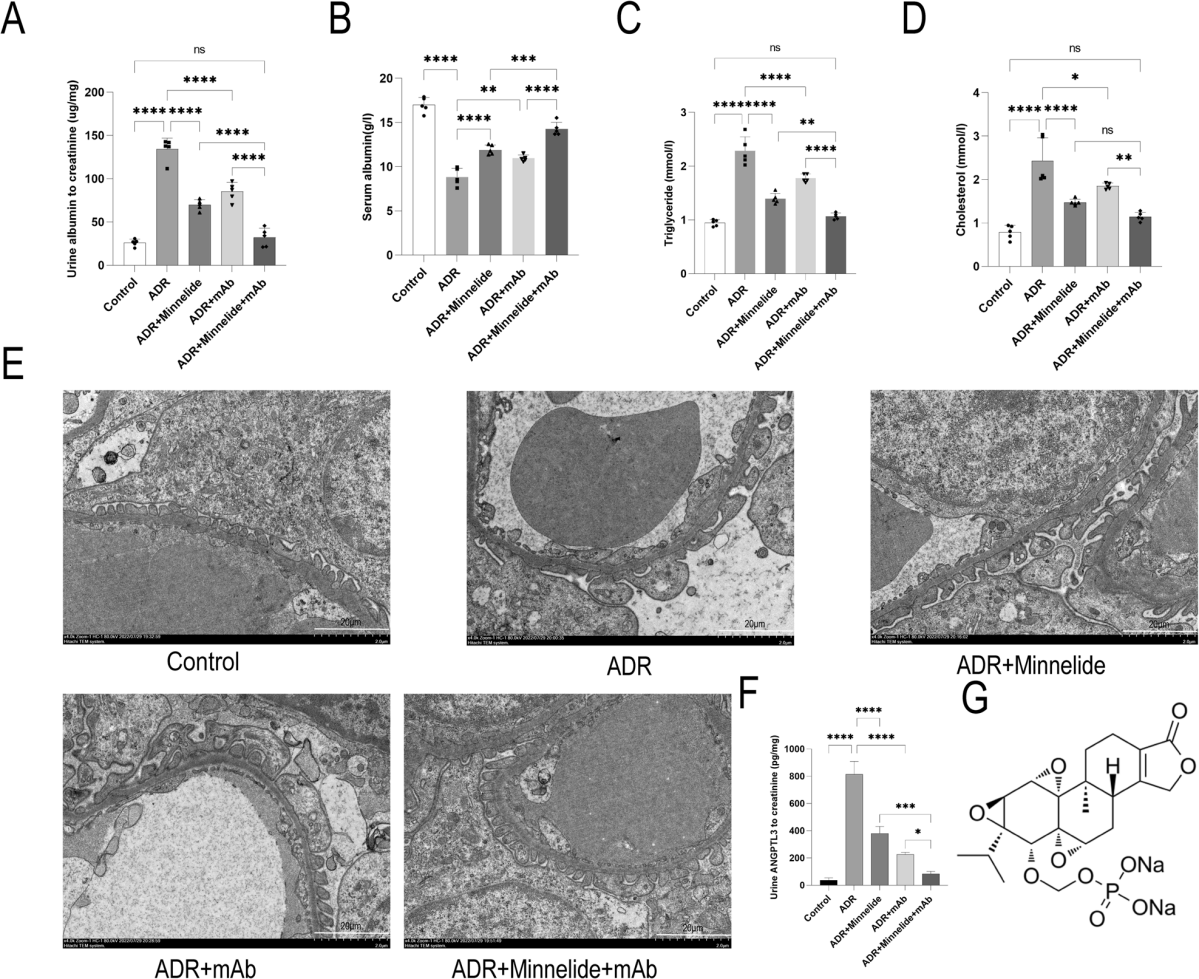
Renal protective effects of minnelide combined with anti-ANGPTL3-FLD monoclonal antibody in mice with AN. **A** Urinary albumin excretion (ug/mg creatinine). **B** Serum albumin level. **C** Serum triglyceride level. **D** Serum cholesterol level. **E** Transmission electron microscopy, bar = 2.0 μm. **F** The expression of ANGPTL3 in urine of mice was detected by Mouse Angiopoietin-like 3 Quantikine ELISA Kit. **G** The structure of minnelide. *n* = 5 ^*^P < 0.05, ^**^P < 0.01, ^***^P < 0.001, and ^****^P < 0.0001.

Compared to the Control group, the urinary albumin creatinine ratio of mice in the ADR group was significantly increased, and either minnelide or mAb alone provided some protection. Surprisingly, the ADR+Minnelide+mAb group achieved complete remission of the urinary albumin creatinine ratio (Fig. 1A). In addition, the serum albumin in the ADR group was significantly reduced compared to the Control group. Minnelide or mAb provided some protection, and the serum albumin was significantly increased in the ADR+Minnelide+mAb group compared to the minnelide or mAb alone treatment groups (Fig. 1B). Similarly, serum triglycerides and cholesterol were significantly elevated in the ADR group compared to the Control, ADR+Minnelide, and the ADR+mAb groups. Nonetheless, triglycerides and cholesterol were significantly decreased in the ADR+Minnelide+mAb group than in the minnelide or mAb alone treatment groups (Fig. 1C, D).

As shown in Fig. 1E, compared to the Control group, foot processes fusion was observed in the ADR group, improved slightly in the ADR+Minnelide and ADR+mAb groups, and completely restored to normal in the ADR+Minnelide+mAb group.

We also examined the expression of ANGPTL3 in the urine of each group of mice and found that urine ANGPTL3 to creatinine ratio was significantly increased in the ADR group compared to the Control group, and either minnelide or mAb treatment alone reduces the expression of ANGPTL3, while it was decreased significantly in the ADR+Minnelide+mAb group compared to the above-mentioned alone treatment groups (Fig. 1F).

### Minnelide combined with anti-ANGPTL3-FLD monoclonal antibody attenuates podocyte injury in mice with AN

**Fig. 2:**
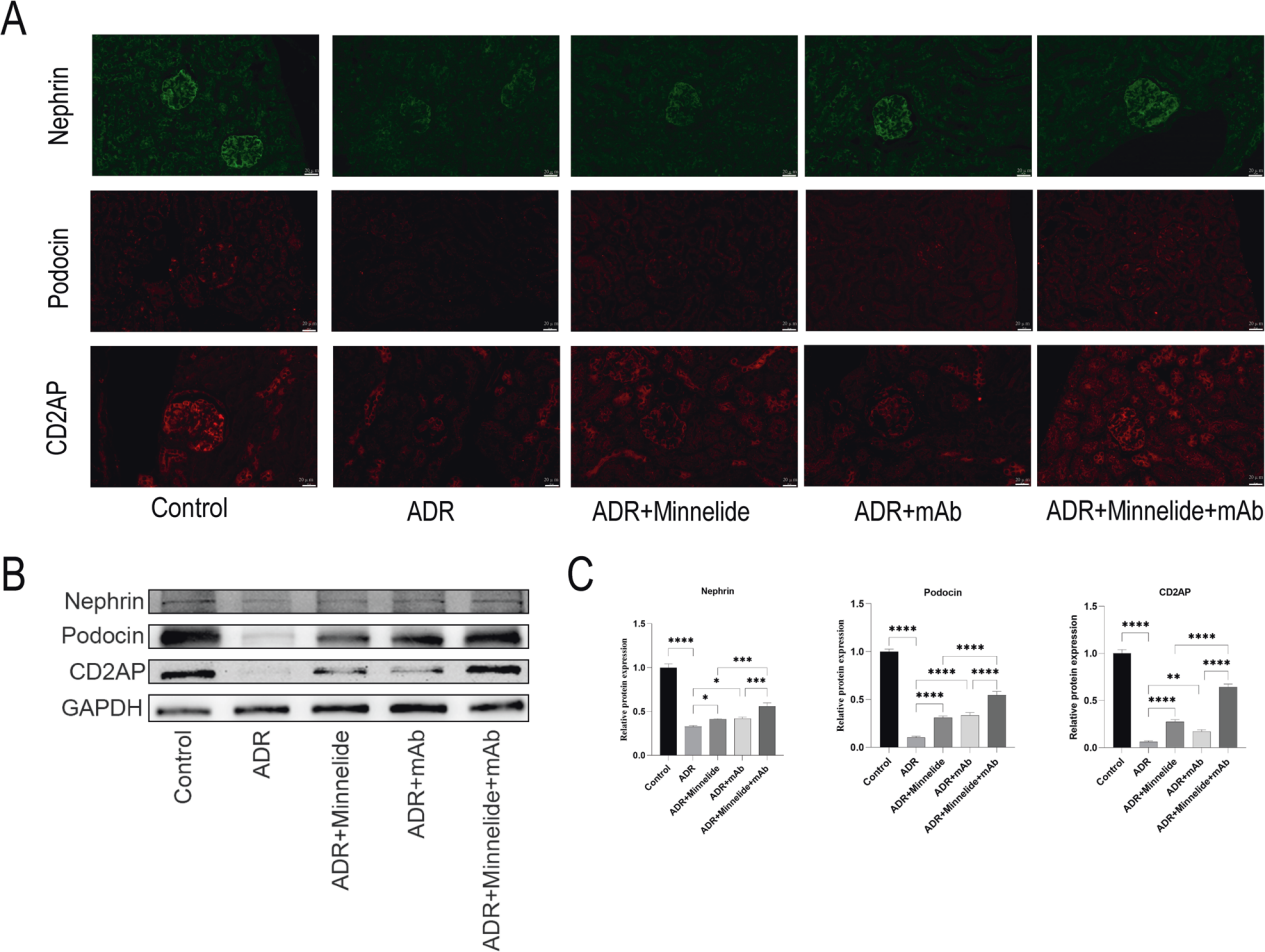
Minnelide combined with anti-ANGPTL3-FLD monoclonal antibody attenuates podocyte injury in mice with AN. **A** Immunofluorescence of Nephrin, Podocin, and Cd2ap in kidney tissues. **B**, **C** The protein expression levels of Nephrin, Podocin, and Cd2ap in kidney tissues were measured by western blot. *n* = 5, ^**^P < 0.01, ^***^P < 0.001, and ^****^P < 0.0001.

The podocyte is a highly differentiated epithelial cell line that is a critical part of the glomerular filtration barrier. Podocyte injury plays a major role in the progression of NS and proteinuria. To investigate whether minnelide combined with anti-ANGPTL3-FLD monoclonal antibody could protect against podocyte injury in mice with AN, we measured the expression of podocyte-associated proteins (Nephrin, Podocin, and CD2AP) in the renal tissues. Compared to the Control, ADR+Minneide, and ADR+mAb groups, the fluorescence intensity of Nephrin, Podocin, and CD2AP was significantly reduced in the ADR group, and that of ADR+Minnelide+mAb group was significantly increased compared to the ADR+Minnelide and ADR+mAb groups (Fig. 2A). Similarly, western blot results demonstrated that the protein levels of Nephrin, Podocin, and CD2AP were significantly decreased in the ADR group than the Control, ADR+Minnelide, and ADR+mAb groups, while those were significantly increased in the ADR+Minnelide+mAb group than in the ADR+Minnelide and ADR+mAb groups (Fig. 2B, C).

### Minnelide combined with anti-ANGPTL3-FLD monoclonal antibody reduces inflammatory factors in mice with AN

**Fig. 3:**
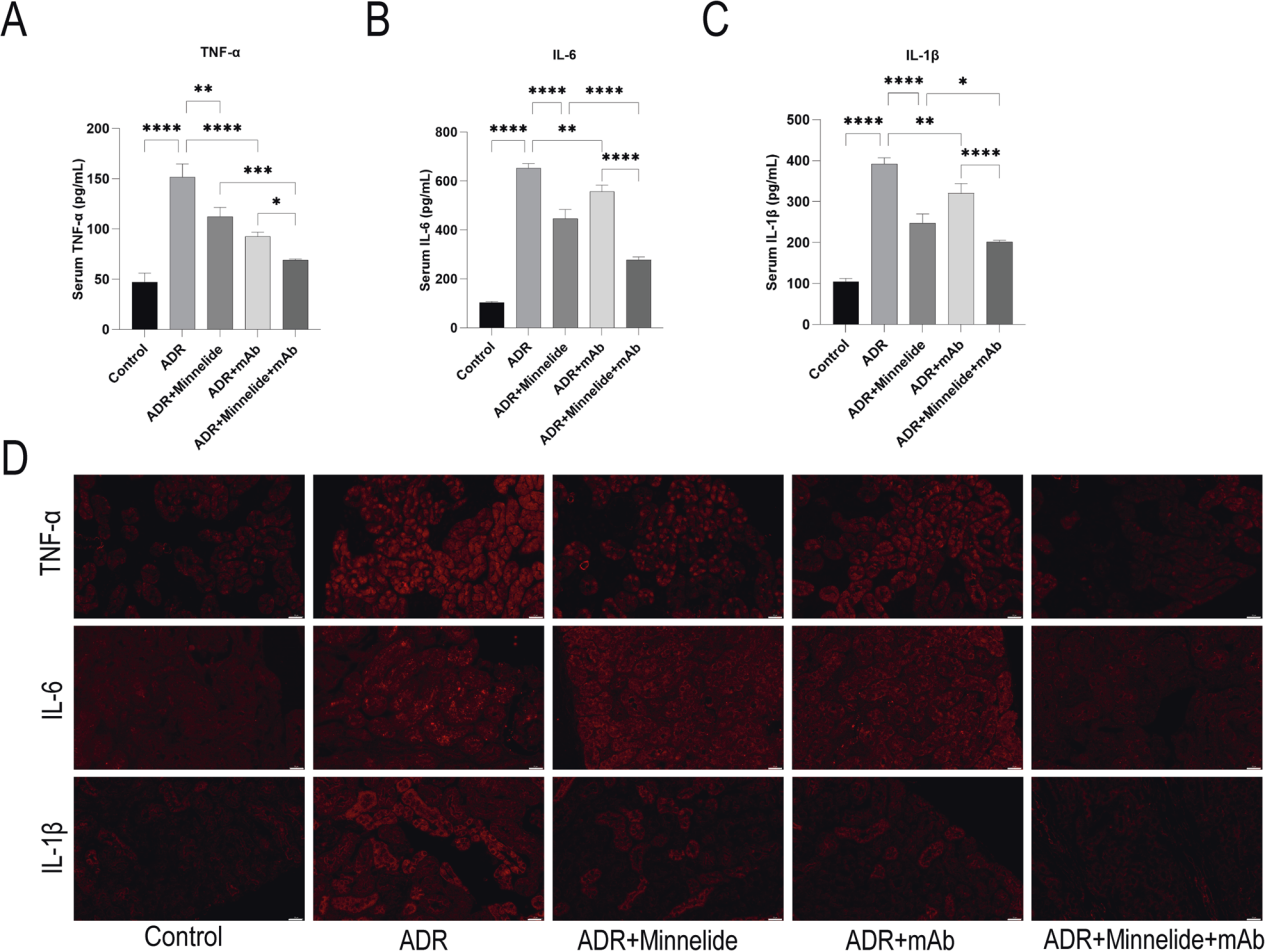
Minnelide combined with anti-ANGPTL3-FLD monoclonal antibody reduces inflammatory factors in mice with AN. **A**–**C** TNF-α, IL-6, and IL-1β were measured in the serum of mice in each group using the ELISA method. **D** Immunofluorescence of TNF-α, IL-6, and IL-1β in renal tissues *n* = 5, ^*^P < 0.05, ^**^P < 0.01, ^***^P < 0.001, and ^****^P < 0.0001.

To further investigate whether minnelide combined with mAb reduces the inflammatory response in mice with AN, we measured the levels of TNF-α, IL-6, and IL-1β in the serum and kidney tissues of mice. The inflammatory factors in mice serum were significantly elevated in the ADR group compared to the Control group, either minnelide or mAb could reduce the level of inflammatory factors. Surprisingly, the level of inflammatory factors in the ADR+Minnelide+mAb group was significantly decreased compared to the above-mentioned alone treatment groups (Fig. 3A–C). Similarly, the fluorescence intensity of inflammatory factors in kidney tissues was significantly increased in the ADR group than in the Control group; either minnelide or mAb reduces the fluorescence intensity of inflammatory factors, and the fluorescence intensity of inflammatory factors in the ADR+Minnelide+mAb group was significantly reduced than the above-mentioned alone treatment groups (Fig. 3D).

### Minnelide combined with anti-ANGPTL3-FLD monoclonal antibody could further alleviate apoptosis in mice with AN

**Fig. 4:**
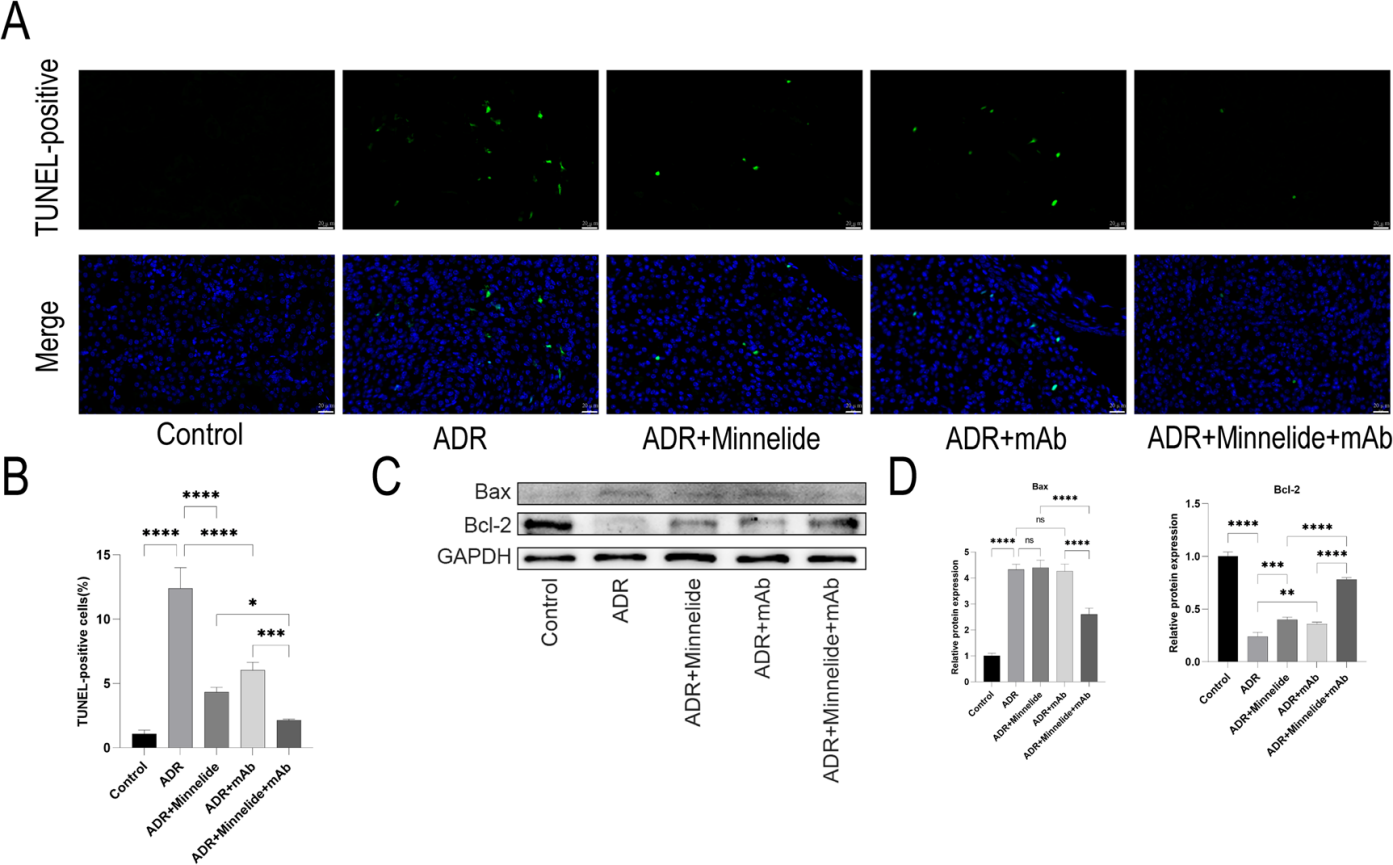
Minnelide combined with anti-ANGPTL3-FLD monoclonal antibody could further alleviate apoptosis in mice with AN. **A**, **B** TUNEL staining of renal tissue in five groups. **C**, **D** Protein expression of Bax and Bcl-2 in five groups of mice are detected by western blot. *n* = 5, ^**^P < 0.01, ^***^P < 0.001, and ^****^P < 0.0001.

To investigate whether minnelide combined with mAb alleviates apoptosis in mice with AN, we examined the apoptosis of kidney tissues in each group of mice by TUNEL staining. The results showed that the apoptotic cells in the ADR group were significantly elevated than those in the Control group, and either minnelide or mAb could alleviate apoptosis. Strikingly, the apoptotic cells in the ADR+Minnelide+mAb group were significantly reduced than that in the above-mentioned alone treatment groups (Fig. 4A, B).

Next, we examined the expression of apoptotic proteins Bax and Bcl-2 by Western blot in the kidney tissues of mice in each group and found that Bax was significantly elevated and Bcl-2 was significantly decreased in the ADR group compared to the Control group; the changes in these proteins were alleviated after treatment with minnelide or mAb alone. Surprisingly, the changes in the above proteins were more relieved in the ADR+Minnelide+mAb group than in the above-mentioned alone treatment groups (Fig. 4C, D).

### Minnelide combined with anti-ANGPTL3-FLD monoclonal antibody could further promote autophagy in mice with AN

**Fig. 5:**
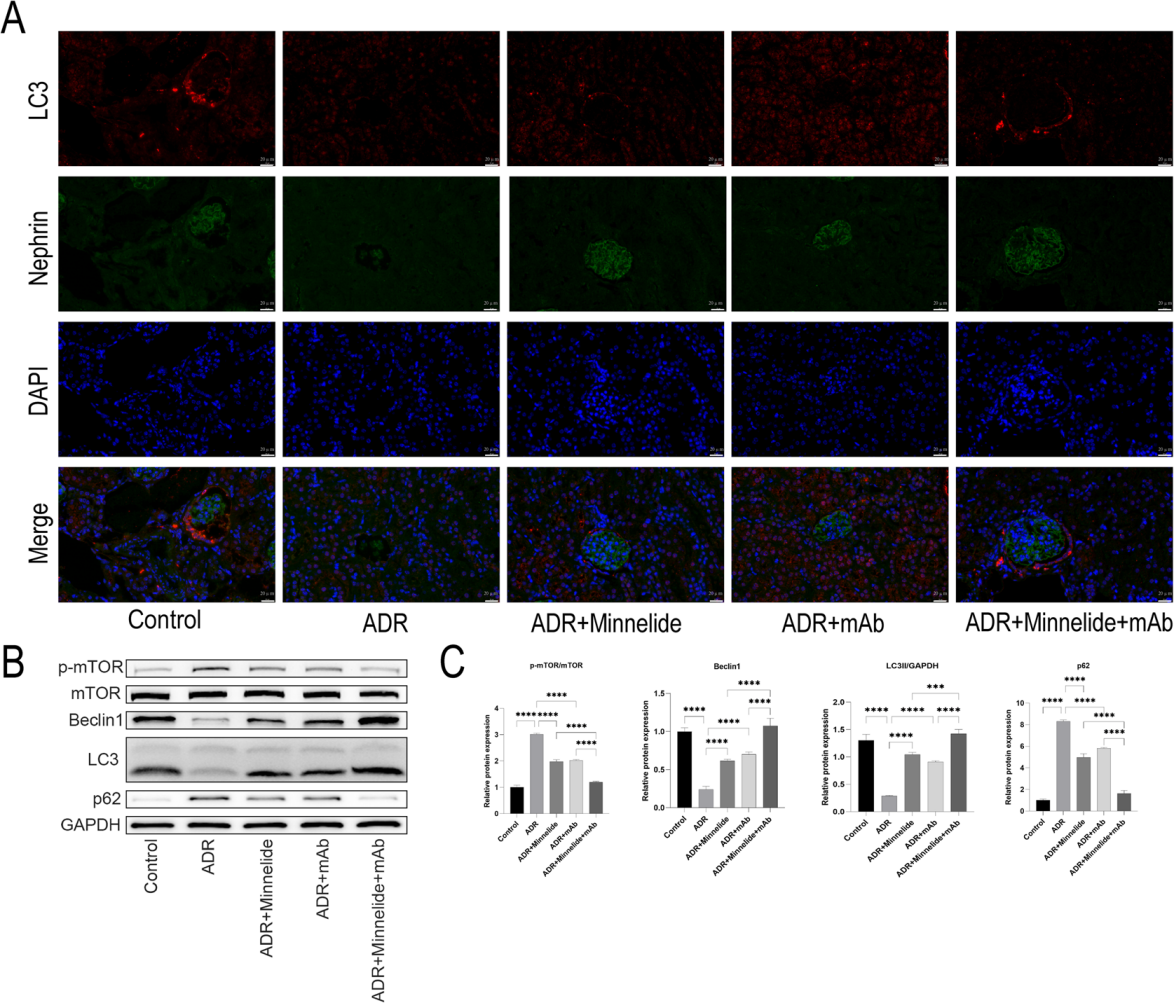
Minnelide combined with anti-ANGPTL3-FLD monoclonal antibody could further promote autophagy in mice with AN. **A** Immunofluorescence staining of LC3 and nephrin. **B**, **C** Protein expression of p-mTOR, mTOR, Beclin1, LC3, and p62 in five groups of mice are detected by western blot. *n* = 5, ^***^P < 0.001 and ^****^P < 0.0001.

Autophagy plays a major role in podocyte homeostasis and function, and autophagy dysfunction is referred to as a marker of podocyte apoptosis in vitro and in vivo [37]. To further investigate whether minnelide combined with mAb protects the podocytes of mice with AN by promoting autophagy, we examined the immunofluorescence and protein expression of the autophagy marker LC3 in kidney tissues in the various groups. The results showed that the fluorescence intensity of LC3 was significantly decreased in the ADR group compared to the Control group, and either minnelide or mAb treatment increased the fluorescence intensity of LC3. Interestingly, the fluorescence intensity of LC3 was significantly increased in the ADR+Minnelide+mAb group than in the treatment group alone (Fig. 5A). Similarly, the protein expression of LC3II in all groups of mice showed a similar trend.

Subsequently, we examined the protein expression of autophagy-related signaling molecules and observed that the expression of both p-mTOR and p62 proteins was significantly elevated in the ADR group, followed by a decrease in autophagy marker protein Beclin1. Conversely, treatment with either minnelide or mAb inhibited the mTOR and p62 signaling pathways and increased the expression of the autophagy marker Beclin1. Moreover, the expression of p-mTOR and p62 proteins in the ADR+Minnelide+mAb group was more reduced, and the autophagy marker protein Beclin1 was more elevated compared to the alone treatment groups (Fig. 5B, C).

### Triptolide combined with anti-ANGPTL3-FLD monoclonal antibody alleviates ADR-induced podocyte injury

**Fig. 6:**
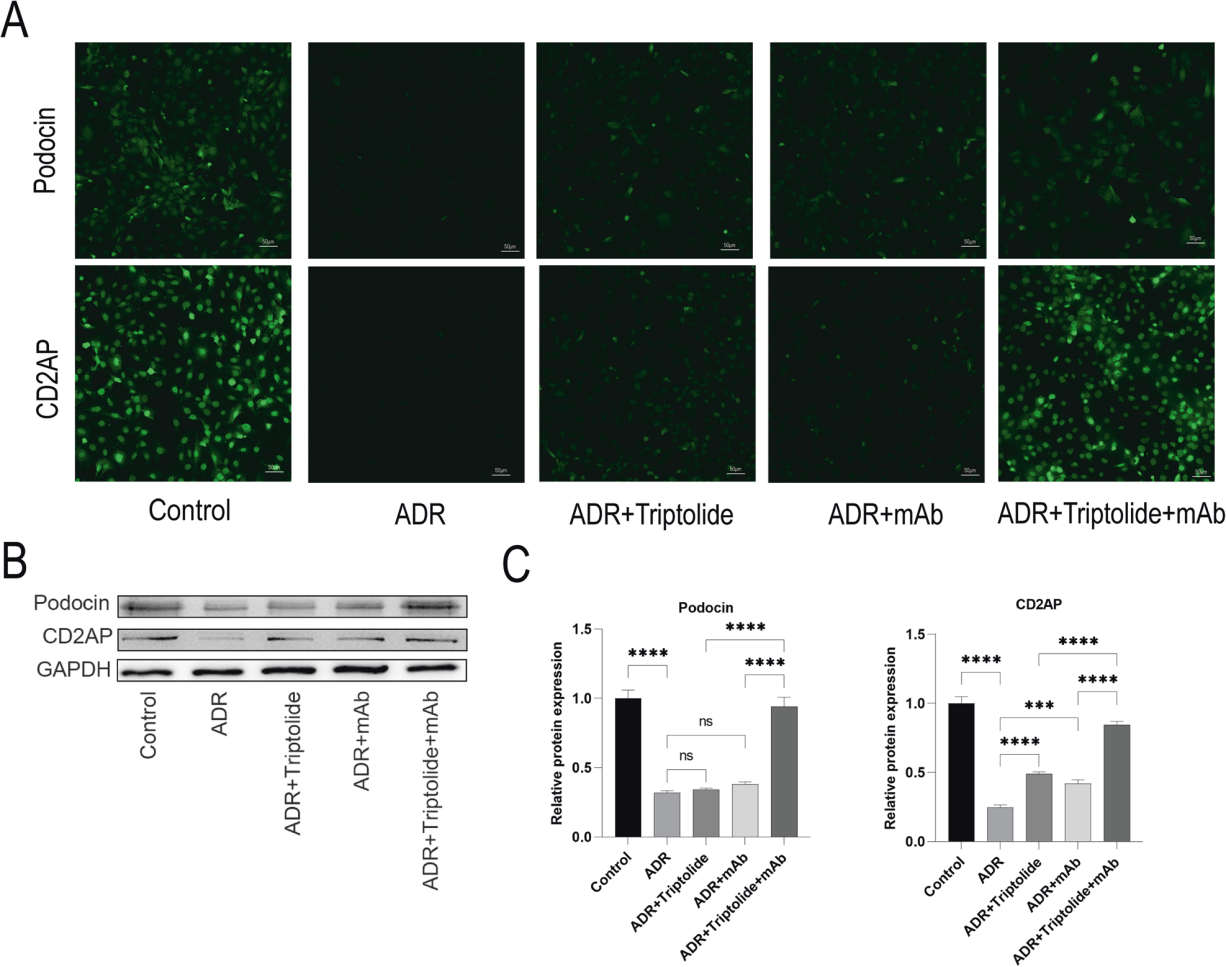
Triptolide combined with anti-ANGPTL3-FLD monoclonal antibody alleviates ADR-induced podocyte injury. **A** Immunofluorescence staining of Podocin and Cd2ap. **B**, **C** The protein expression of Podocin and Cd2ap in podocytes was measured by western blot. *n* = 5, ^***^P < 0.001 and ^****^P < 0.0001.

To further investigate whether triptolide combined with mAb has a protective effect against ADR-induced podocyte damage, we examined the expression of podocyte-associated proteins (Podocin and CD2AP) in podocytes. Compared to the Control group, the fluorescence intensities of these proteins were significantly decreased in the ADR group. Treatment with either triptolide or mAb enhanced the protein expression to some extent, and the fluorescence intensity of the ADR+Triptolide+mAb group was significantly increased compared to the above-mentioned alone treatment groups (Fig. 6A).

Next, we employed a Western blot and found that the protein levels of Podocin and CD2AP showed a similar trend (Fig. 6B, C).

### Triptolide combined with anti-ANGPTL3-FLD monoclonal antibody alleviates ADR-induced podocyte apoptosis

**Fig. 7:**
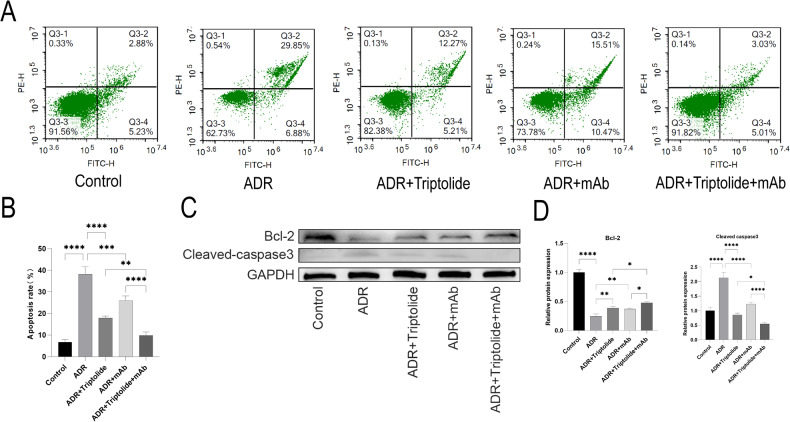
Triptolide combined with anti-ANGPTL3-FLD monoclonal antibody alleviates ADR-induced podocyte apoptosis. **A**, **B** PE–Annexin-V staining followed by FCM demonstrated that ADR-induced apoptosis was alleviated by triptolide or mAb in podocytes. **C**, **D** The protein expression of Bcl-2 and Cleaved caspase3 in podocytes was measured by western blot. *n* = 5, ^*^P < 0.05, ^**^P < 0.01^,^ and ^****^P < 0.0001.

To further investigate whether triptolide combined with mAb alleviates ADR-induced podocyte apoptosis, we detected the apoptosis rate of each group of podocytes by flow cytometry. The results showed that the apoptosis rate was significantly increased in the ADR group compared to the Control group, and either triptolide or mAb could alleviate apoptosis, and the apoptosis rate in the ADR+Triptolide+mAb group was remarkably reduced than in the above-mentioned alone treatment groups(Fig. 7A, B).

Next, we detected the protein levels of Bcl-2 and Cleaved caspase3 by Western blot and found that Bcl-2 expression was significantly decreased and Cleaved caspase3 expression was significantly increased in the ADR group, which was relieved slightly by treatment with either triptolide or mAb. Interestingly, the protein levels of Bcl-2 and Cleaved caspase3 were significantly alleviated in the ADR+Triptolide+mAb group than in the above-mentioned treatment alone groups (Fig. 7C, D).

### Triptolide combined with anti-ANGPTL3-FLD monoclonal antibody promotes autography in ADR-induced podocytes

**Fig. 8:**
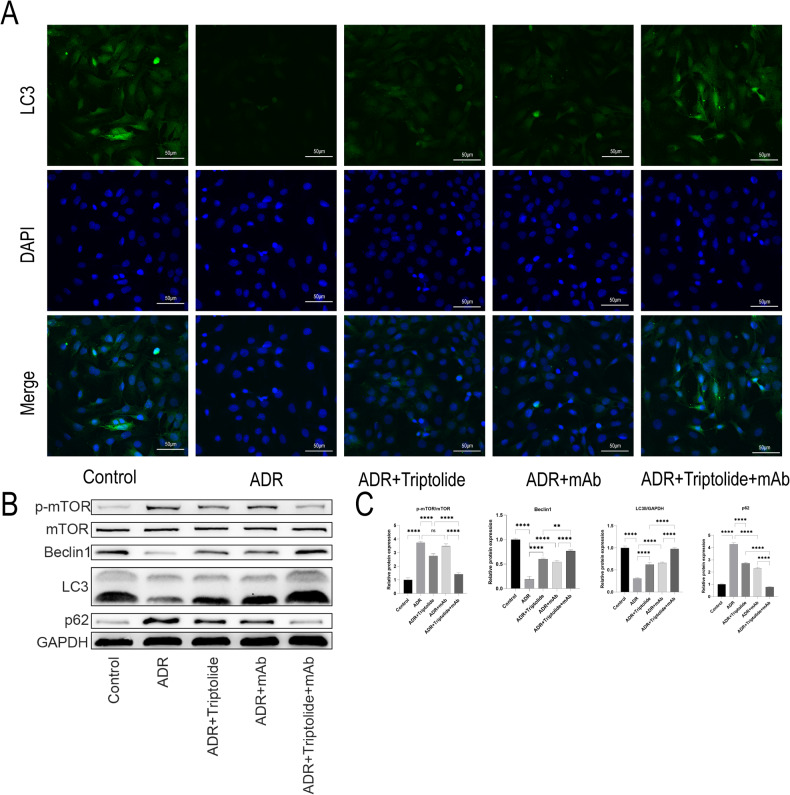
Triptolide combined with anti-ANGPTL3-FLD monoclonal antibody promotes autography in ADR-induced podocytes. **A** Immunofluorescence staining of LC3 in podocytes. **B**, **C** The protein expression of p-mTOR, mTOR, Beclin1, LC3, and p62 in five groups of podocytes was detected by western blot. *n* = 5, ^**^P < 0.01 and ^****^P < 0.0001.

To further investigate whether triptolide combined with mAb protects against ADR-induced podocyte injury by promoting autophagy in vitro, we examined the immunofluorescence and protein level of LC3, a marker of autophagy, in different groups of podocytes. The results showed that the fluorescence intensity of LC3 was significantly decreased in the ADR group compared to the Control group, and either triptolide or mAb could improve the fluorescence intensity of LC3. Surprisingly, the fluorescence intensity of the ADR+Triptolide+mAb group was significantly increased compared to the alone treatment groups (Fig. 8A). Similarly, the protein level of LC3II was similar in all groups of podocytes.

Moreover, mTOR signaling and p62 levels are key regulators of autophagy. Next, we examined mTOR signaling and p62 levels by Western blot and found that the protein level was significantly increased and autophagy marker Beclin1 was significantly decreased in the ADR group, and the treatment with either triptolide or mAb alone alleviated the effect. Also, the above protein changes were relieved to a greater extent in the ADR+Triptolide+mAb group compared to the treatment groups alone (Fig. 8B, C).

### Triptolide combined with anti-ANGPTL3-FLD monoclonal antibody restored autophagic flux and increased the number of autophagosomes in ADR-induced podocytes injury

**Fig. 9:**
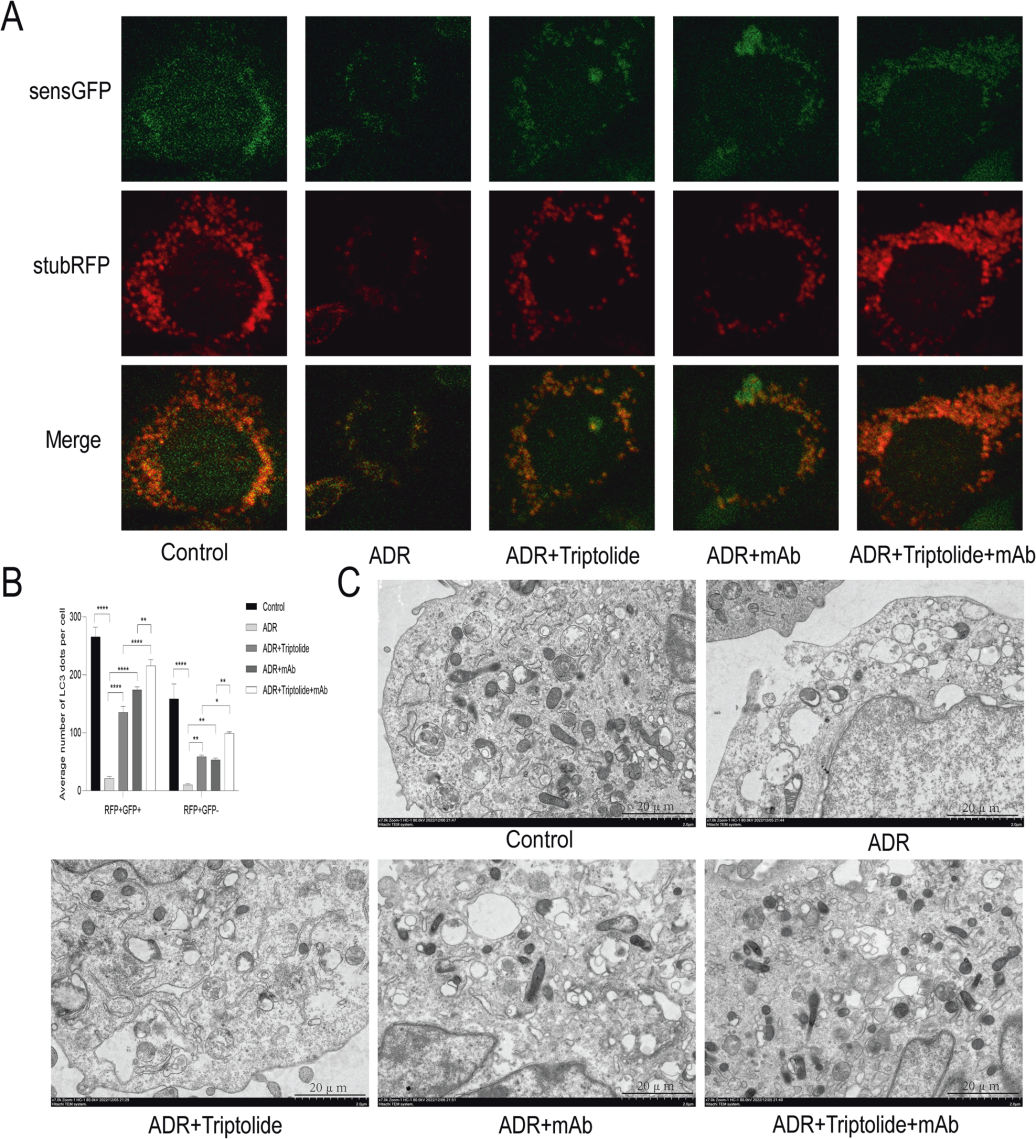
Triptolide combined with anti-ANGPTL3-FLD monoclonal antibody restored autophagic flux and increased the number of autophagosomes in ADR-induced podocytes injury. **A**, **B** The changes of autophagic flux in different treatment groups. **C** Assessment of autophagosomes by transmission electron microscopy, bar = 2.0 μm. *n* = 5, *P < 0.05, **P < 0.01, and ****P < 0.0001.

To further explore the effects of triptolide and mAb on autophagic flux, we examined the changes of autophagic flux in different groups by infecting mouse podocytes with GFP-RFP-LC3 adenovirus. The results showed that compared to the Control group, ADR treatment significantly reduced the red and yellow dots, and either triptolide or mAb treatment increased the red and yellow dots; the yellow and red dots were increased further in the ADR+Triptolide+mAb group (Fig. 9A, B).

In addition, we observed the effect of triptolide combined with mAb on the number of autophagosomes in podocytes by TEM. The results showed a large number of autophagosomes in the cytoplasm of the podocytes in the Control group, a significant decrease in autophagosomes after ADR treatment, and a specific increase in autophagosomes after treatment with triptolide or mAb alone. Intriguingly, the number of autophagosomes in the ADR+Triptolide+mAb group was significantly higher than in the above-mentioned alone treatment groups (Fig. 9C).

### The effects of minnelide on the liver, ovaries and testes

**Fig. 10:**
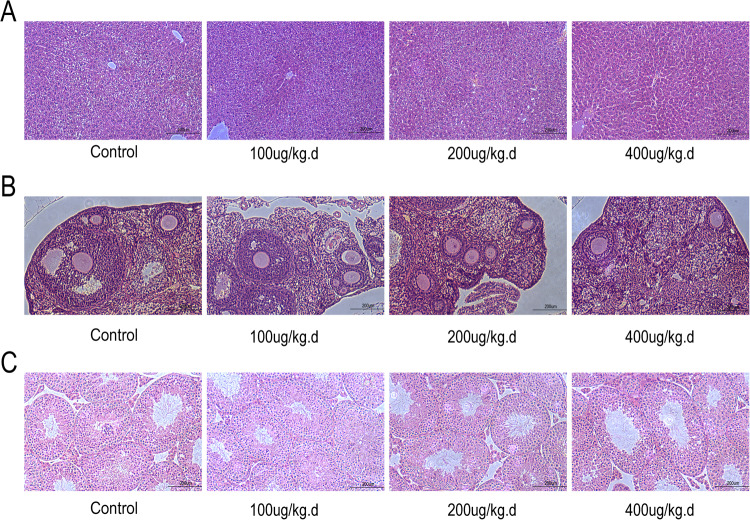
The effects of minnelide on the liver, ovaries and testes. **A** Effect of different doses of minnelide on liver pathology. **B** Effect of different doses of minnelide on Ovaries pathology. **C** Effect of different doses of minnelide on testes pathology.

To investigate the toxicity of minnelide on mice liver and reproductive system, different doses (0, 100, 200, and 400 μg/kg.d) were injected intraperitoneally for 1 week. The results showed that either of the doses had no significant effects on the liver, ovaries, and testes of mice (Fig. 10A–C).

## Discussion

MCD is the most common type of NS in children, and the long-term use of glucocorticoids and immunosuppressive drugs has many side effects and does not alleviate proteinuria in some patients [1, 2, 42]. Therefore, there is an urgent need for improved methods for NS treatment while avoiding the side effects of drugs. In the present study, either minnelide or mAb alleviated proteinuria, and minnelide combined with mAb almost completely attenuated proteinuria and the ultrastructure of podocytes. In vitro, triptolide combined with mAb provided greater protection against ADR-induced podocyte injury compared to treatment alone. Thus, minnelide combined with mAb could be a novel approach to completely alleviate the proteinuria of NS.

Highly differentiated podocytes are a major part of the glomerular filtration barrier, and podocyte injury is closely associated with the progression of many kidney diseases [43–45]. Adriamycin nephropathy mice are a classical NS mouse model with the fusion of processes on electron microscopy in vivo [46, 47]. Moreover, ADR induces apoptosis, skeleton rearrangement, and downregulation of slit diaphragm protein expression (Nephrin, Podocin, and CD2AP) in podocytes in vitro [32, 41, 48]. In the present study, AN and ADR-induced podocyte injury mice were used as the model to study the protective effect of minnelide or mAb on podocyte damage in vivo and in vitro.

Angptl3 is closely related to lipid metabolism, but its correlation with the kidney is yet unclear [49, 50]. The current study found that Angptl3 is closely related to the development of NS, and Angptl3 is a major factor involved in PAN or ADR-induced podocyte injury [20, 51]. In AN mice, Angptl3 knockout could largely alleviate podocyte injury and reduce proteinuria [22]. Moreover, mAb could also provide some protection in AN mice, however, proteinuria is still detected [52]. Triptolide, the main active ingredient of traditional Chinese medicine, has been used in China for >40 years for the treatment of glomerular diseases [53, 54]. Minnelide, a water-soluble prodrug of triptolide, has been used in phase II clinical trials for the treatment of cancer [26, 29, 55]. The present study showed that minnelide combined with mAb reduced proteinuria in mice with AN and elevated the levels of Nephrin, Podocin, and CD2AP protein expression compared to treatment alone in vivo. Similarly, triptolide combined with mAb further increased the expression of Nephrin, Podocin, and CD2AP in ADR-induced podocyte injury compared to alone treatment in vitro.

Inflammation is closely related to the pathophysiology of kidney disease, and regulating inflammation is a critical approach to slowing down the progression of kidney disease [56–58]. Therefore, we tested the levels of inflammatory factors in the serum and renal tissues of different treatment groups and found that either minnelide or mAb alone reduces the inflammatory factor levels compared to the ADR group, while minnelide combined with mAb reduces the inflammatory factor levels to a greater extent compared to alone treatments.

In renal diseases, the decrease in the number of podocytes is closely related to the progression of renal diseases [59, 60]. Podocyte apoptosis is the primary cause of podocyte loss in glomerular diseases, and alleviating podocyte apoptosis would increase the number of the nephron in the glomerulus, thus contributing to the protection of renal function [45, 61, 62]. Therefore, we detected apoptosis in mice and podocytes of different treatment groups. Compared to the Control group, more TUNEL-positive cells were detected in the ADR group; either minnelide or mAb could attenuate the number of apoptotic cells, and minnelide combined with mAb could attenuate the apoptotic cells to a large extent. In addition, Western blot showed that Bax and Cleaved caspase3 protein levels were significantly increased, and Bcl-2 protein expression was significantly decreased in the ADR group. These alterations could be alleviated by the treatment with either minnelide or mAb to some extent but markedly by combination treatment in vivo. Similarly, flow cytometry and Western blot detection of Bcl-2 and Cleaved caspase3 found similar trends in vitro.

Autophagy is a recycling system that provides energy for organelle renovation by degrading damaged organelles or proteins inside the cell [63, 64]. Podocyte autophagy helps maintain homeostasis and function, and autophagy induction can reduce podocyte injury [36, 38, 65]. Therefore, we examined autophagy and its signaling pathways in each treatment group and found that both autophagy markers LC3 and Beclin1 were significantly decreased in the ADR group. Minnelide or mAb increases their expression to some extent, and minnelide combined with mAb further increases the protein expression compared to the alone treatment group. In addition, the expression of mTOR signaling pathway proteins, p-mTOR and p62, was significantly increased in the ADR group and was reduced by either minnelide or mAb; minnelide combined with mAb further reduced their expression compared to the alone treatment group. Similarly, autophagy and its signaling pathways to the podocytes also reflected the same trend in vitro.

Hepatic, renal, and reproductive toxicity is the main obstacle to the clinical application of triptolide [26, 28, 66]. However, the toxicity of triptolide is time- and dose-dependent, and short-time application of small doses is safe [28, 30, 67]. To investigate the hepatotoxicity and reproductive toxicity of minnelide, we injected different doses of minnelide intraperitoneally into female and male mice for 1 week. The pathological results did not show any significant effects of different doses of minnelide on the liver, ovaries, and testes.

In conclusion, minnelide combined with mAb almost completely protected AN mice by promoting autophagy and inhibiting apoptosis in podocytes. Thus, minnelide combined with mAb would provide an ideal approach for NS treatment. Because of the toxicity of minnelide, in the future, it is hoped that the antibody can be optimized and the dose of minnelide can be reduced based on ensuring complete relief of proteinuria, which will lay the groundwork for later clinical application.

### Reporting summary

Further information on research design is available in the Nature Research Reporting Summary linked to this article.

## Supplementary information

Reporting Summary

Original western blots

## Data Availability

Data will be made available on request.

## Declarations

All mice experiments were approved by the Ethics Committee of Fudan University (approval number: IDM2021055).
