# Supplementary material for: Minnelide combined with anti-ANGPTL3-FLD monoclonal antibody completely protects mice with adriamycin nephropathy by promoting autophagy and inhibiting apoptosis
Source: Cell Death Dis. 2023 Sep 9;14(9):601. doi: 10.1038/s41419-023-06124-0 (PMC10492865; doi:10.1038/s41419-023-06124-0)

Figure 2

Nephrin


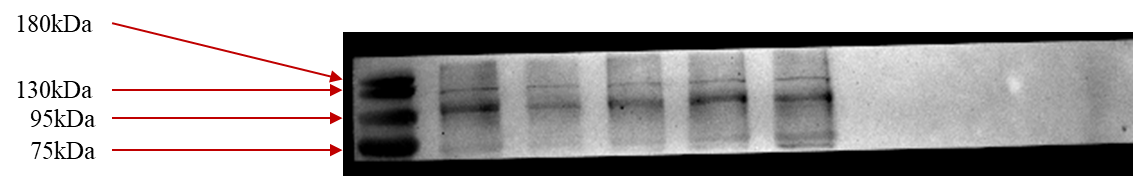


Podocin


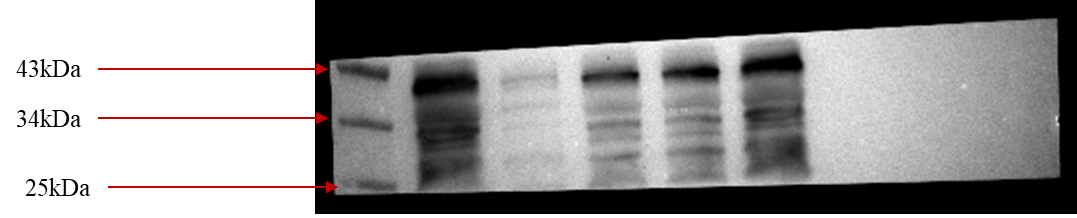


CD2AP


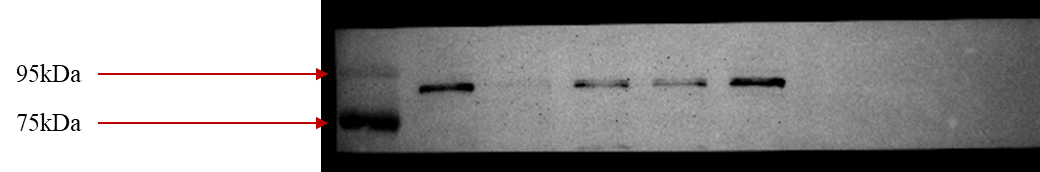


GAPDH


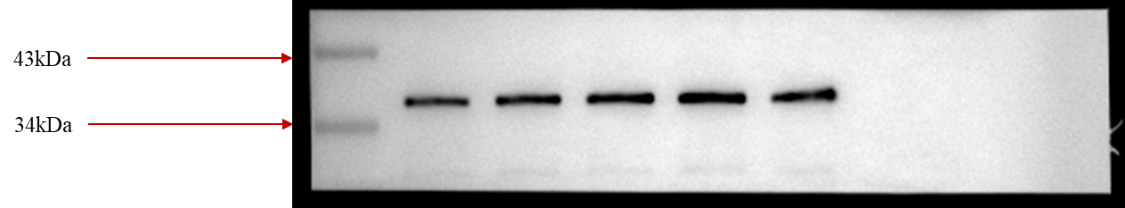


Figure 4

Bax


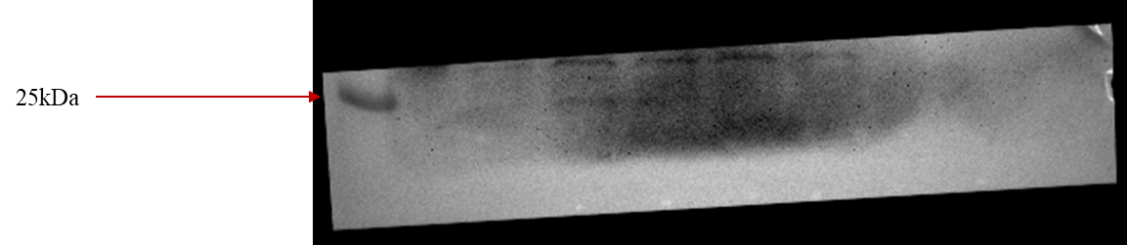


Bcl-2


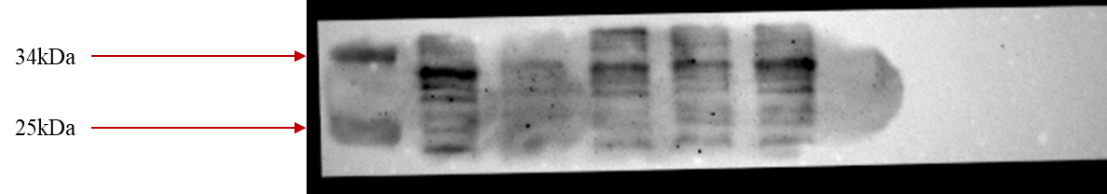


GAPDH


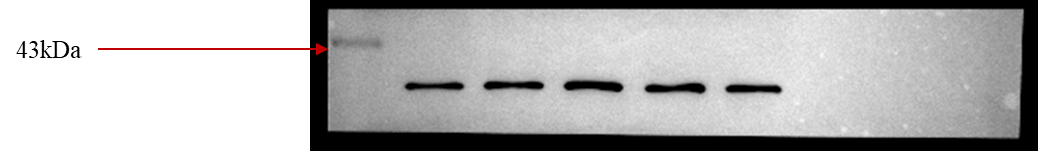


Figure 5

p-mTOR


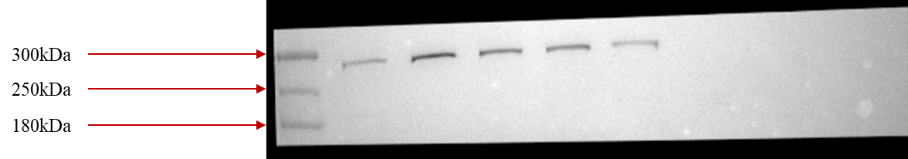


mTOR


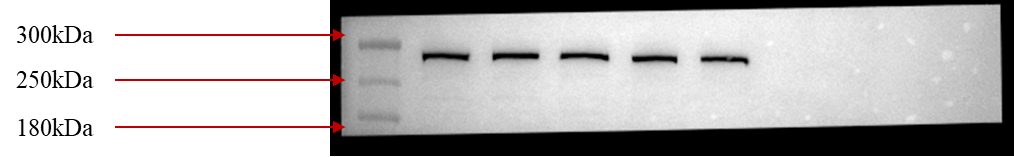


Beclin1


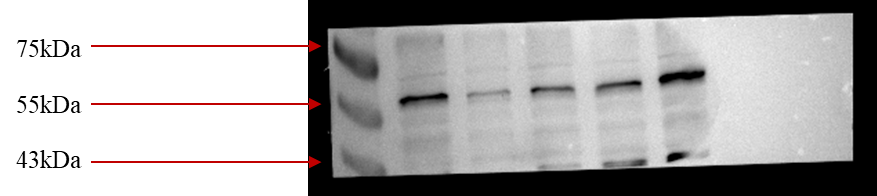


LC3


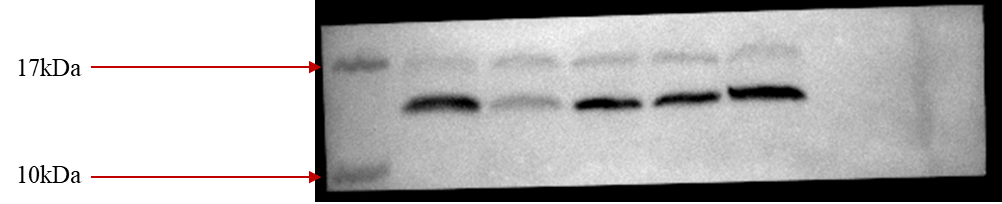


P62


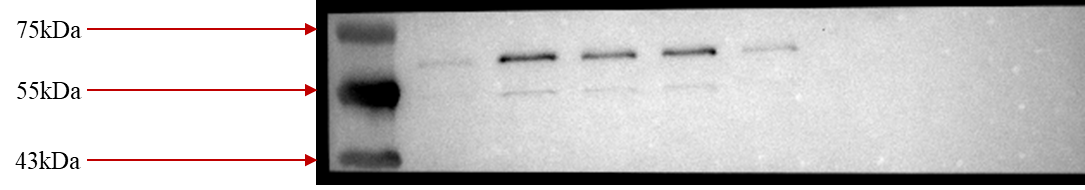


GAPDH


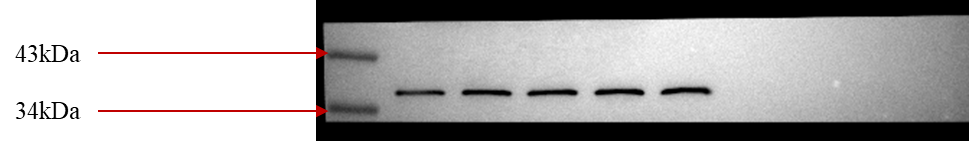


Figure 6

Podocin


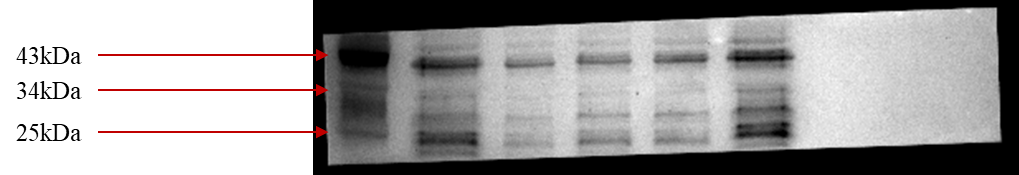


CD2AP


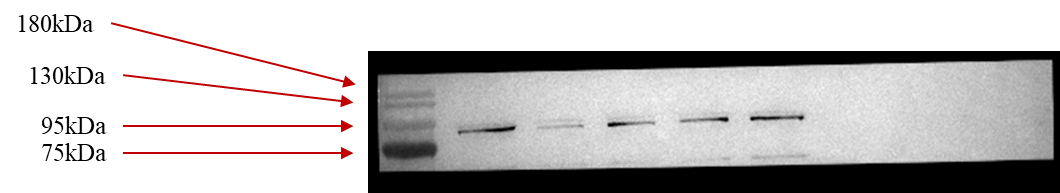


GAPDH


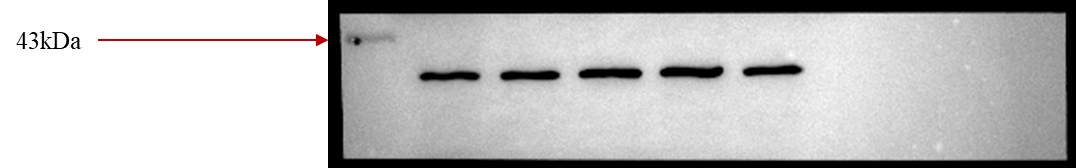


Figure 7

Bcl-2


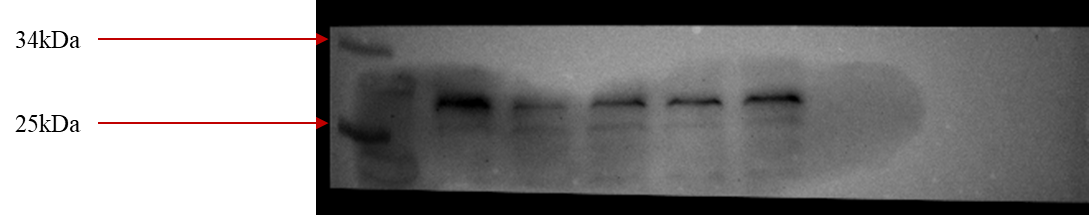


Cleaved caspase 3


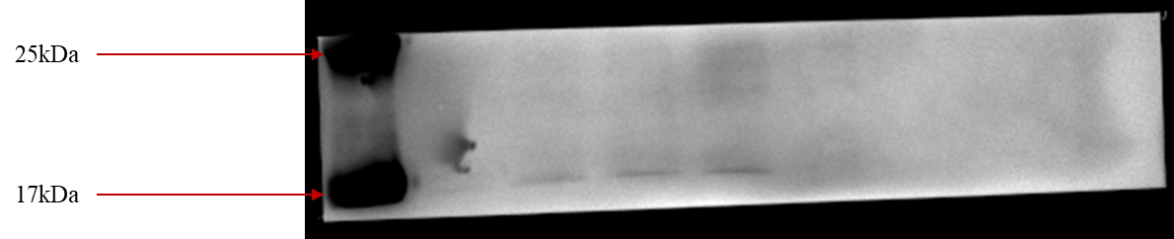


GAPDH


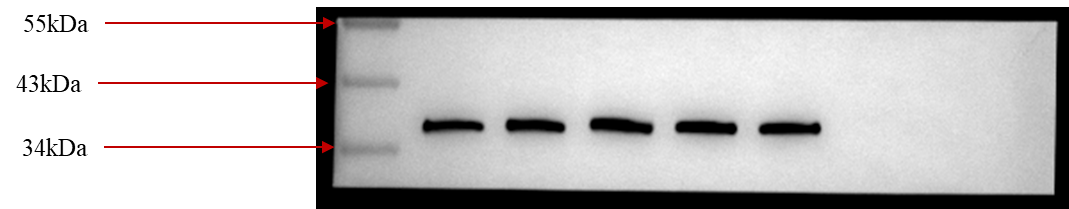


Figure 8

p-mTOR


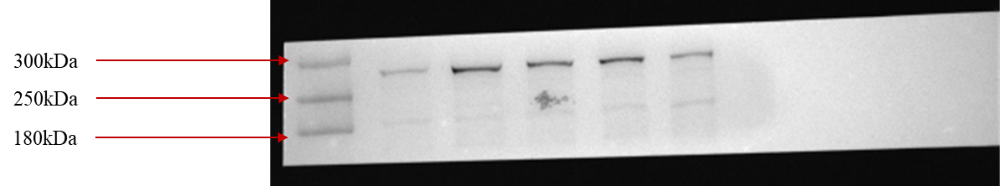


mTOR


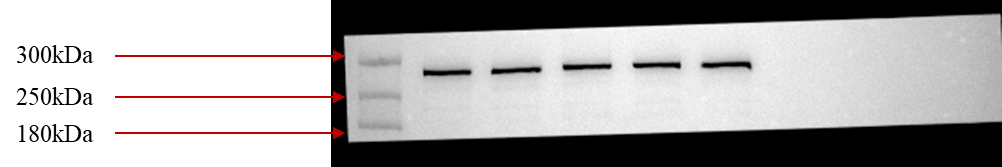


Beclin1


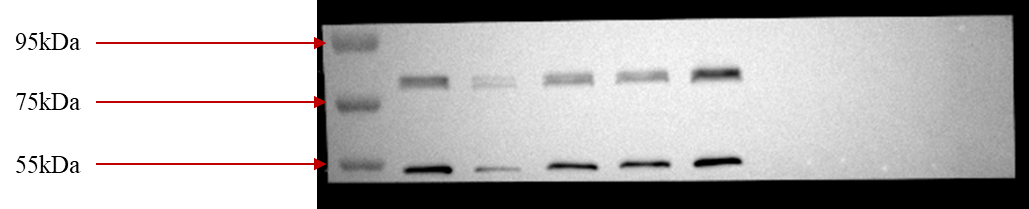


LC3


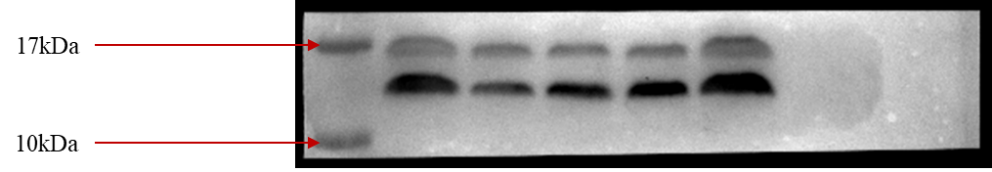


P62


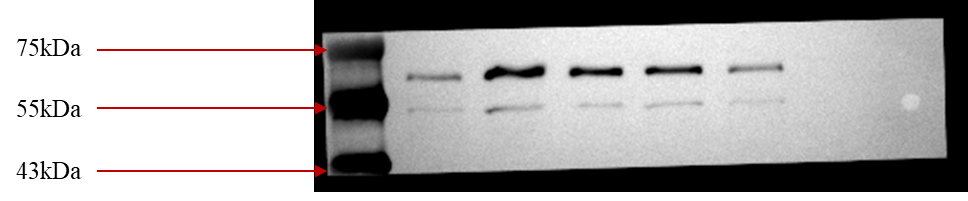


GAPDH


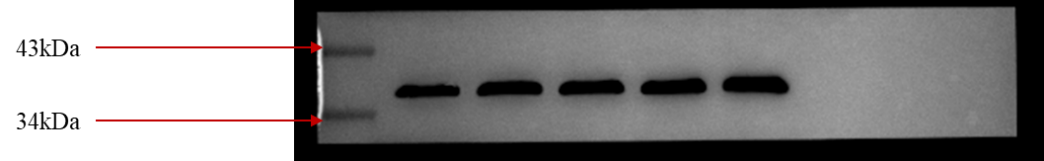

Supplement: Supplementary file 2 — Original western blots [file 41419_2023_6124_MOESM2_ESM.docx]
